# Mechanism of luteolin in treating chronic heart failure by regulating HIF-1 signaling pathway-mediated mitochondrial damage to inhibit cardiomyocyte apoptosis

**DOI:** 10.3389/fcell.2026.1977166

**Published:** 2026-10-02

**Authors:** Weiqi Shi, Zhide Zhu, Junshen Lu, Meiling Tang, Yan Pang, Zhilin Peng, Jie Lu, Puwei Huang, Shanyun Zou, Jiaji Wu, Yarou Tu, Chunmei Fan, Yuyun Gan, Lihui Hu, Qi Lin, Liuqin Qin, Rusheng Shi, Fei Zhong, Yuxuan Shen, Jianqi Lu

**Affiliations:** 1 The First College of Clinical Medicine, Guangxi University of Chinese Medicine, Nanning, Guangxi, China; 2 Affiliated Traditional Chinese Medicine School of Guangxi University of Chinese Medicine, Nanning, Guangxi, China; 3 The First Affiliated Hospital of Guangxi University of Chinese Medicine, Nanning, Guangxi, China

**Keywords:** cardiomyocyte apoptosis, chronic heart failure, HIF-1 signaling pathway, luteolin, mitochondrial damage

## Abstract

**Background:**

Chronic heart failure (CHF) is a systemic cardiovascular disease characterized by an extremely poor prognosis. Luteolin demonstrates promising therapeutic potential in the prevention and management of CHF. This study aims to explore the mechanism by which luteolin ameliorates CHF through mitigating mitochondrial damage and inhibiting cardiomyocyte apoptosis, using bioinformatics analysis combined with *in vitro* and *in vivo* experiments.

**Methods:**

Network pharmacology was employed to identify functional targets of luteolin, as well as target genes associated with CHF and mitochondrial damage. The overlapping targets from the three datasets were obtained to construct a protein-protein interaction (PPI) network. Gene Ontology (GO) and Kyoto Encyclopedia of Genes and Genomes (KEGG) enrichment analyses, followed by molecular docking, were then conducted. Bioinformatic approaches were utilized to screen potential signature genes from the PPI network to build a clinical prediction model. Single-cell transcriptomics (scRNA-seq) was performed to characterize the expression profiles of these signature genes in cardiomyocyte subpopulations. Lastly, the results were comprehensively validated by *in vitro* and *in vivo* experiments.

**Results:**

In total, 329 overlapping targets of luteolin, mitochondrial damage and CHF were obtained; 122 core targets with above-average degree values were mainly enriched in the HIF-1 pathway. Six signature genes (HIF1A, NOS2, BNIP3, BNIP3L, PDK1, and BBC3; collectively termed the L–M–C gene set) were identified through 113 machine-learning combinations. The six-gene clinical model demonstrated favorable predictive performance. scRNA-seq demonstrated progressive upregulation of L-M-C genes during the transition from normal to injured cardiomyocytes in CHF. *In vitro*, luteolin markedly downregulated L-M-C genes (*P* < 0.05) and attenuated doxorubicin-induced cardiomyocyte injury. *In vivo*, in CHF rats, luteolin reduced myocardial L-M-C gene expression (*P* < 0.05), ameliorated myocardial lesions, and suppressed fibrosis and cardiomyocyte apoptosis.

**Conclusion:**

Luteolin exerts therapeutic effects against CHF by inhibiting the HIF-1 signaling pathway and downregulating the expression of target genes such as NOS2 and BNIP3. This regulatory action attenuates myocardial mitochondrial damage and fibrosis, thereby ameliorating the pathological structural remodeling of the myocardium.

## 1 Introduction

Chronic heart failure (CHF) is a systemic cardiovascular disorder characterized by structural and functional impairment of the myocardium, diminished ventricular pumping capacity, and subsequent systemic circulatory dysfunction. It is notorious for its extremely poor prognosis, alongside high morbidity and mortality rates (Butler et al., 2025; Boulin et al., 2026; Roger, 2021). The deleterious consequences of CHF are severe, often manifesting as reduced exercise tolerance and a progressively declining quality of life. This not only exacerbates the physical and psychological burden on patients but also imposes substantial medical and economic strains on families and healthcare systems (Global Burden of Cardiovascular Diseases and Risks 2023 Collaborators; Darvish et al., 2025).

The maintenance of mitochondrial functional homeostasis is critical for sustaining the normal metabolism of cardiomyocytes and overall cardiac functional equilibrium (Lopaschuk et al., 2021). Throughout the onset and progression of CHF, widespread mitochondrial damage and loss of homeostasis occur within cardiomyocytes. The disruption of mitochondrial structure and function can trigger cardiomyocyte apoptosis. This continuous apoptotic loss of cardiomyocytes is a primary driver of the deterioration of myocardial contractility and the exacerbation of ventricular remodeling in heart failure (Da Dalt et al., 2023). The HIF-1 signaling pathway serves as one of the central pathways regulating myocardial mitochondrial injury, oxidative stress, and cellular metabolism. Within this pathway, HIF1A acts as the core oxygen-sensitive subunit, exerting pivotal regulatory roles in myocardial hypoxic injury, mitochondrial dysfunction, cardiomyocyte apoptosis, and cardiac remodeling (Raj et al., 2025; Sant'Ana et al., 2023; Chaillou, 2018). Aberrant activation of HIF1A can intensify myocardial mitochondrial damage, thereby inducing cardiomyocyte apoptosis and promoting myocardial fibrosis. Conversely, suppressing the hyperactivation of the HIF-1 signaling pathway can effectively ameliorate mitochondrial impairment, inhibit cardiomyocyte apoptosis, and consequently delay the progression of CHF (Packer, 2020; Wang et al., 2025).

Luteolin, a natural flavonoid compound widely distributed across various medicinal herbs, possesses robust pharmacological properties, including anti-inflammatory, antioxidant, and cytoprotective capacities (Muruganathan et al., 2022; Xu et al., 2020; Pan et al., 2022; Ren et al., 2024). Recently, research on luteolin in the context of cardiovascular disease prevention and treatment has accumulated. Multiple studies have corroborated its ability to alleviate myocardial injury, attenuate cardiac remodeling, and preserve myocardial energy metabolism, collectively highlighting its substantial therapeutic potential for CHF (Wang et al., 2023; Zhu et al., 2024). Nevertheless, the precise molecular mechanisms by which luteolin modulates the HIF-1 signaling pathway to ameliorate mitochondrial damage, inhibit cardiomyocyte apoptosis, and subsequently treat CHF remain incompletely elucidated. In the present study, we applied integrated bioinformatics approaches, complemented by comprehensive *in vitro* and *in vivo* experimental validations, to explore the mechanism of luteolin in treating CHF via the targeted regulation of HIF1A to alleviate myocardial mitochondrial injury and suppress apoptosis. This study aims to provide a robust scientific rationale for the clinical application of natural flavonoid agents in the prevention and management of CHF.

## 2 Materials and methods

### 2.1 Prediction of target genes for Luteolin, mitochondrial damage, and CHF

The structural data file (SDF) of luteolin was retrieved from the PubChem database (https://pubchem.ncbi.nlm.nih.gov) and submitted to the SwissTargetPrediction platform (version 2019; www.swisstargetprediction.ch) and PharmMapper server (version 2017; http://www.lilab-ecust.cn/pharmmapper) on May 15, 2026, with the species parameter set to *Homo sapiens* for the prediction of putative pharmacological targets. All predicted targets were downloaded directly without applying a probability cutoff. Targets predicted by PharmMapper were initially reported as UniProt IDs and subsequently converted to official gene symbols using the UniProt ID mapping tool (https://www.uniprot.org/). The target lists from both platforms were merged in Microsoft Excel and deduplicated by gene symbol, yielding the definitive pharmacological target profile of luteolin. Separately, target genes associated with mitochondrial damage (search keyword: “mitochondrial damage”) and chronic heart failure (CHF; search keyword: “chronic heart failure”) were retrieved from the GeneCards database (Version 6.1, https://www.genecards.org) on May 15, 2026. The intersection of the luteolin target set and the two disease-related gene sets was determined by matching gene symbols, resulting in the final intersection gene set.

### 2.2 Acquisition of intersecting genes and construction of the protein-protein interaction (PPI) network

The identified pharmacological targets of luteolin, along with the disease-associated targets for mitochondrial damage and CHF, were imported into the OmicShare bioinformatics platform (https://www.omicshare.com/) to identify their overlapping genes. A PPI network of these intersecting targets was then constructed using the STRING database (https://www.string-db.org). Unconnected nodes were hidden, and the network was exported to Cytoscape (version 3.8.0) for topological analysis. Targets with degree values equal to or above the median were designated as core targets, and a refined PPI network was subsequently visualized.

### 2.3 Gene ontology (GO) and Kyoto encyclopedia of genes and genomes (KEGG) enrichment analyses and molecular docking

The targets from the PPI network were subjected to GO functional annotation and KEGG pathway enrichment analysis using the OmicShare tool. For GO enrichment, the top 10 categories from each of the three GO domains (biological process, molecular function, and cellular component) were selected for visualization. Similarly, the top 30 significantly enriched pathways from KEGG analysis were visualized. Furthermore, core targets with degree values ≥80 in the PPI network were subjected to molecular docking simulations with luteolin.

### 2.4 Screening of the “luteolin-mitochondrial damage-CHF” (L-M-C) model gene set

The GSE9128, GSE59867, GSE21125, and GSE11947 datasets were downloaded from the Gene Expression Omnibus (GEO) database (https://www.ncbi.nlm.nih.gov/geo/). Microarray gene expression data were standardized and adjusted using the limma package (version 3.4.0) in R. Based on the core target genes derived from the PPI network, 12 distinct machine learning algorithms were applied to generate 113 unique algorithmic combinations, to identify an L-M-C gene set characterized by optimal accuracy and stability. The algorithms included Least Absolute Shrinkage and Selection Operator (Lasso), Ridge regression, Stepwise Generalized Linear Model (Stepglm), Extreme Gradient Boosting (XGBoost), Random Forest (RF), Elastic Net (Enet), Partial Least Squares Regression for Generalized Linear Models (plsRglm), Generalized Boosted Regression Modeling (GBM), Naive Bayes, Linear Discriminant Analysis (LDA), Boosted Generalized Linear Models (glmBoost), and Support Vector Machine (SVM). The area under the receiver operating characteristic curve (AUC) was calculated for each algorithm combination, and the model yielding the highest average AUC was selected as the optimal model. A PPI network and a correlation heatmap of expression profiles were generated for the finalized model gene set to assess the association and co-expression among the genes in the L-M-C signature.

### 2.5 Assessment of model gene set goodness-of-fit

The GSE9128 dataset was used as the training cohort, while the GSE59867, GSE21125, and GSE11947 datasets were integrated to form an independent validation cohort. To evaluate the predictive robustness of the L-M-C gene set, a nomogram was constructed using regression modeling methodologies. Based on the data from both the training and validation sets, receiver operating characteristic (ROC) and calibration curves were plotted to evaluate the model’s diagnostic performance. Moreover, Decision Curve Analysis (DCA) and Clinical Impact Curves (CIC) were performed to evaluate the net clinical benefit.

### 2.6 Processing of scRNA-seq data

The single-cell RNA sequencing (scRNA-seq) dataset was obtained from the Gene Expression Omnibus (GEO) database with accession number GSE222144 (GPL24676 platform). All samples were of human origin (*H. sapiens*). The dataset included one cardiac sample from a heart failure patient (GSM6915590; HF group) and one normal cardiac sample (GSM6915591; control group). The raw count matrix was converted into a Seurat object using the CreateSeuratObject function in the Seurat package of R (version 4.4.1), and the Harmony algorithm was applied for batch effect correction across samples. Dimensionality reduction and clustering were performed using the FindNeighbors and FindClusters functions, followed by identification of marker genes for each cluster using the FindAllMarkers function. Each cell cluster was annotated for cell type based on the expression profiles of canonical cell-type markers.

### 2.7 Cellular proportions, gene set scoring, and KEGG enrichment analysis

Following cell cluster annotation, the proportion of each cell type relative to the total cell population across samples was calculated. The module score of the L-M-C gene set in each cell subpopulation was quantified using the AddModuleScore function in the Seurat package, termed the “L-M-C score”.

### 2.8 Pseudotime trajectory analysis and dynamic gene identification

Single-cell lineage trajectory inference was performed using the Monocle3 package in R. The cell subpopulation with the highest L-M-C score was isolated, and dimensionality reduction was performed using the DDRTree algorithm to construct the cellular differentiation trajectory. Cells in the S1 state were defined as the developmental root, and the resulting trajectory was visualized. The expression profiles of all genes in the L-M-C signature were extracted to visualize their expression dynamics along pseudotime.

### 2.9 Cell culture and CCK-8 assay

The rat cardiomyocyte cell line H9C2 was purchased from Wuhan Procell Life Science and Technology Co., Ltd (Wuhan, China) and cultured in the manufacturer’s complete medium for H9C2 cells (Procell, Wuhan, China). The cells were maintained in a humidified incubator at 37 °C under a 5% CO_2_ atmosphere (Yiheng Instruments, Shanghai, China). To evaluate the concentration-dependent effect of luteolin (Yuanye Bio-Technology, Shanghai, China) on cell viability, a Cell Counting Kit-8 (CCK-8) assay was performed. H9C2 cells were treated with various concentrations of luteolin (5 μmol/L, 10 μmol/L, 15 μmol/L, and 20 μmol/L) for 24, 48, and 72 h. The highest concentration of luteolin that showed no significant cytotoxicity at 72 h was selected as the optimal concentration for subsequent experiments.

### 2.10 Cell grouping and intervention protocols

The cultured H9C2 cells were randomly assigned to four groups: Control group, Model group, Inhibitor group, and Luteolin group. The Control group was maintained in the standard complete medium. To construct an *in vitro* model of cardiomyocyte injury, the Model group was treated with complete medium containing 1 μmol/L doxorubicin (Yuanye Bio-Technology, Shanghai, China) for 24 h (Zhang et al., 2024). On the basis of the injury model, the Inhibitor group was co-treated with 10 μmol/L PX-478 (TargetMol Chemicals Inc., Boston, MA, United States), a known HIF-1 inhibitor (Chang et al., 2022). Similarly, the Luteolin group was treated with the optimal concentration of luteolin (as determined by CCK-8 assay) in combination with doxorubicin.

### 2.11 Immunofluorescence assays

H9C2 cells in all experimental groups were successively fixed, permeabilized, and blocked. Cells were then incubated overnight at 4 °C with an anti-HIF1A primary antibody (HuaAn Biotechnology, Hangzhou, China). The next day, a fluorophore-conjugated secondary antibody (Servicebio, Wuhan, China) and DAPI nuclear counterstain (Servicebio, Wuhan, China) were added sequentially in the dark. Fluorescence images were captured using an inverted fluorescence microscope (Nikon, Tokyo, Japan), and the mean fluorescence intensity of HIF1A was quantified among groups.

### 2.12 Toluidine blue and sirius red staining

Following fixation, permeabilization, and blocking, H9C2 cells were stained using a Toluidine Blue Staining Kit (Servicebio, Wuhan, China) (Luo et al., 2021) and a Sirius Red Staining Kit (Servicebio, Wuhan, China) (She et al., 2025), respectively. The staining characteristics and morphological changes of the cells were observed and recorded under a light microscope (Nikon, Tokyo, Japan).

### 2.13 RT-qPCR analysis

Total RNA isolation from H9C2 cells was performed on ice using the TRNzol Universal RNA extraction reagent (Servicebio, Wuhan, China). Subsequent reverse transcription and cDNA amplification were performed using the following thermal cycling protocol: an initial denaturation at 95 °C for 15 min, followed by 40 cycles of denaturation at 95 °C for 10 s, annealing at 60 °C for 30 s, and extension at 72 °C for 1 min, with a final extension at 72 °C for 2 min. All reactions were performed in biological triplicate. Raw data were normalized to the endogenous control GAPDH, and the relative gene expression was calculated using the 2^−ΔΔCT^ method. Primer sequences and corresponding amplicon sizes (Servicebio, Wuhan, China) are listed in Supplementary Table S1.

### 2.14 Animal grouping and construction of the CHF model

Forty-eight specific pathogen-free (SPF) grade Sprague-Dawley (SD) rats (280–300 g, balanced sex ratio) were purchased from Hunan Slike Jingda Experimental Animal Co., Ltd (License: SCXK (Xiang) 2021-0002, Animal Certificate No.430727251101793137). The study protocol was ethically approved (approval No. GXTCMU-EC KS20260515-28). Following a one-week acclimatization period, the rats were allocated into four groups using a random number table method (n = 12 per group): Control, Model, Sacubitril/Valsartan, and Luteolin groups. The Control rats underwent a sham procedure involving a thoracotomy and suturing without coronary artery occlusion. In contrast, the Model, Sacubitril/Valsartan, and Luteolin groups underwent left anterior descending (LAD) coronary artery ligation to establish the CHF model (Pfeffer et al., 1985). Six weeks after surgery, five rats were randomly selected from each group for echocardiographic assessment under isoflurane inhalation anesthesia. After examination, the rats were returned to their original groups, and all 12 rats per group were subsequently used for histological and Western blotting analyses. No mortality occurred during or after surgery, and all LAD-ligated rats met the left ventricular ejection fraction (LVEF) ≤ 40% criterion with no exclusions (Figure 1, Supplementary Table S2).

**FIGURE 1:**
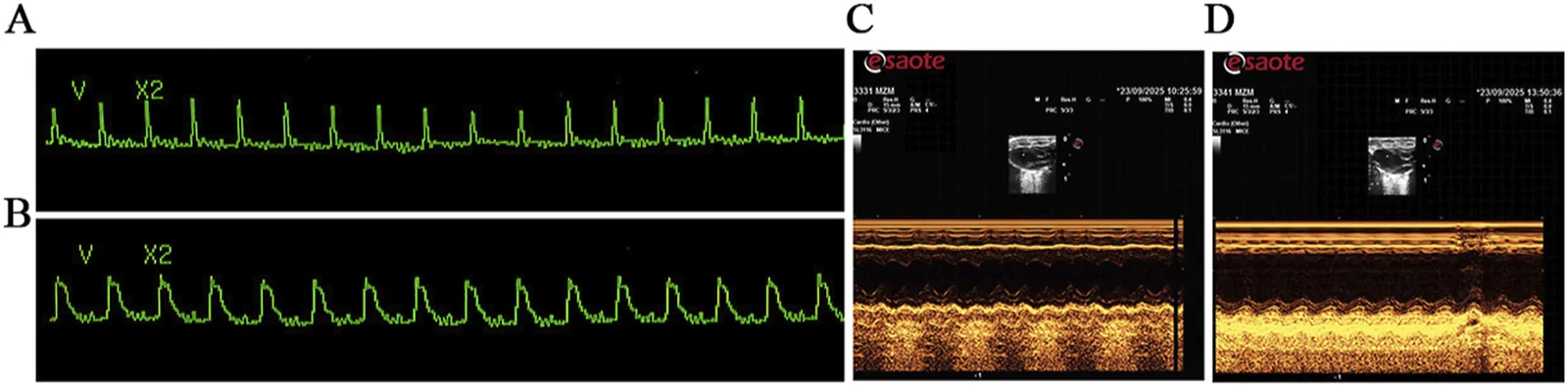
Electrocardiograms during surgery and postoperative echocardiograms of rats in the Control and Model groups. **(A)** Electrocardiogram of a Control group rat during surgery (thoracotomy and suture only, without coronary artery ligation). **(B)** Electrocardiogram of a Model group rat during surgery (thoracotomy with ligation of the left anterior descending coronary artery). **(C)** M-mode echocardiogram of a Control group rat. **(D)** M-mode echocardiogram of a Model group rat, showing increased left ventricular internal diameter and decreased wall motion.

### 2.15 *In vivo* interventions and tissue harvesting

Treatment began the day after successful model establishment was confirmed, with drugs administered via daily oral gavage for 28 consecutive days. Both the Control and Model groups received the same volume of physiological saline. The Luteolin group was administered a luteolin-saline solution at a dose of 30 mg/kg/d and a volume of 4 mL/kg (Nakayama et al., 2015). The positive control group received sacubitril/valsartan at 68 mg/kg/d, suspended in a 0.5% CMC-Na physiological saline vehicle, at the same volume of 4 mL/kg (Nordén et al., 2025). At the end of the treatment period, the rats were euthanized by inhalation of high-concentration isoflurane (fresh oxygen flow at 1 L/min, with the vaporizer set to 5% isoflurane, continuous inhalation for 5–8 min) (Chisholm and Pang, 2016). Cardiac tissues were excised, fixed in 4% paraformaldehyde, dehydrated, and embedded in paraffin for subsequent sectioning.

### 2.16 Histological staining procedures

Paraffin-embedded myocardial tissue blocks were sectioned and deparaffinized. The sections were stained with hematoxylin and eosin (H&E) (Servicebio, Wuhan, China), Masson’s trichrome (Beyotime, Shanghai, China), and Sirius red (Servicebio, Wuhan, China). Sections were examined microscopically to assess myocardial structure, the extent of collagen deposition, and the severity of interstitial fibrosis.

### 2.17 Transmission electron microscopy (TEM)

Fresh myocardial tissue segments (1 mm^3^) were rapidly excised and prefixed in 2.5% glutaraldehyde at 4 °C, followed by a secondary fixation in 1% osmium tetroxide at room temperature. The specimens were then dehydrated through a graded series and embedded in epoxy resin. Ultrathin sections were double-stained with uranyl acetate and lead citrate, allowing visualization of mitochondrial morphology, membrane integrity, and cristae structure under a transmission electron microscope.

### 2.18 Western blot assay

Cultured H9C2 cells were lysed and myocardial tissues were homogenized, followed by total protein extraction. Protein concentrations in all samples were quantified using a BCA protein assay kit. After equal protein loading, the protein lysates were separated by SDS-PAGE, transferred onto membranes, and blocked. The blots were then incubated overnight at 4 °C with primary antibodies against HIF1A, NOS2, BNIP3, BNIP3L, PDK1, and BBC3 (HuaAn Biotechnology, Hangzhou, China). After incubation with corresponding secondary antibodies for 1 h at room temperature, chemiluminescent detection was performed. Target protein bands were quantified by densitometry and normalized to the internal control β-actin, with band intensity analyzed using Gel-Pro Analyzer version 4.0 software.

### 2.19 TUNEL staining

Deparaffinized myocardial tissue sections were stained using a TUNEL apoptosis detection kit (Servicebio, Wuhan, China) strictly according to the manufacturer’s instructions. The sections were examined under an inverted fluorescence microscope to quantify the proportion of TUNEL-positive nuclei as a measure of cardiomyocyte apoptosis.

### 2.20 Immunofluorescence detection in tissues

Following deparaffinization and 20 min fixation in paraformaldehyde, the myocardial tissue sections were permeabilized using Triton X-100. Non-specific binding sites were blocked with 5% bovine serum albumin (BSA) blocking buffer. The sections were subsequently incubated overnight at 4 °C with primary antibodies against HIF1A, Bax, Bcl-2, and Caspase-3 (HuaAn Biotechnology, Hangzhou, China). The next day, fluorophore-conjugated secondary antibodies (Servicebio, Wuhan, China) and DAPI nuclear stain were added at room temperature, in the dark. After mounting with anti-fade mounting medium, images were captured using a fluorescence microscope to analyze expression patterns of HIF1A and key apoptosis-related proteins.

### 2.21 Statistical analysis

All statistical analyses were performed using SPSS version 22.0 software (IBM Corp., Armonk, NY, United States), and graphs were generated using GraphPad Prism 9 software (GraphPad Software, La Jolla, CA, United States). Quantitative data were expressed as mean ± standard deviation (x̄ ± s). Normality was assessed using the Shapiro-Wilk test, and homogeneity of variance was evaluated using Levene’s test. All datasets were normally distributed with equal variances (all *P* > 0.05). For comparisons among multiple groups, one-way analysis of variance (ANOVA) was performed, followed by Tukey’s HSD *post hoc* test for pairwise comparisons. Statistical significance was defined as *P* < 0.05.

## 3 Results

### 3.1 Network pharmacology analysis and molecular docking results

A total of 351 pharmacological target genes for luteolin were identified, while 13,500 and 14,924 genes were associated with mitochondrial damage and CHF, respectively. The intersection of these three sets yielded 329 common targets (Figure 2A). Among these, 122 targets had degree values above the mean and were thus defined as core targets (Figure 2B). In the GO functional enrichment analysis, these core targets were mainly enriched in biological processes such as the regulation of programmed cell death, programmed cell death, and general cell death. Enriched molecular functions included catalytic activity and anion binding, while cellular components were mainly enriched in the cytosol and vesicle lumen (Figures 2D–F). Furthermore, the HIF-1 signaling pathway was ranked second in KEGG analysis among the top 30 significantly enriched pathways (Figure 2C). For proteins with degree ≥80 in the PPI network—namely AKT1 (degree = 82), HIF1A (degree = 81), HSP90AA1 (degree = 80), and EGFR (degree = 80)—molecular docking simulations revealed excellent binding affinities with luteolin (Figure 2G).

**FIGURE 2:**
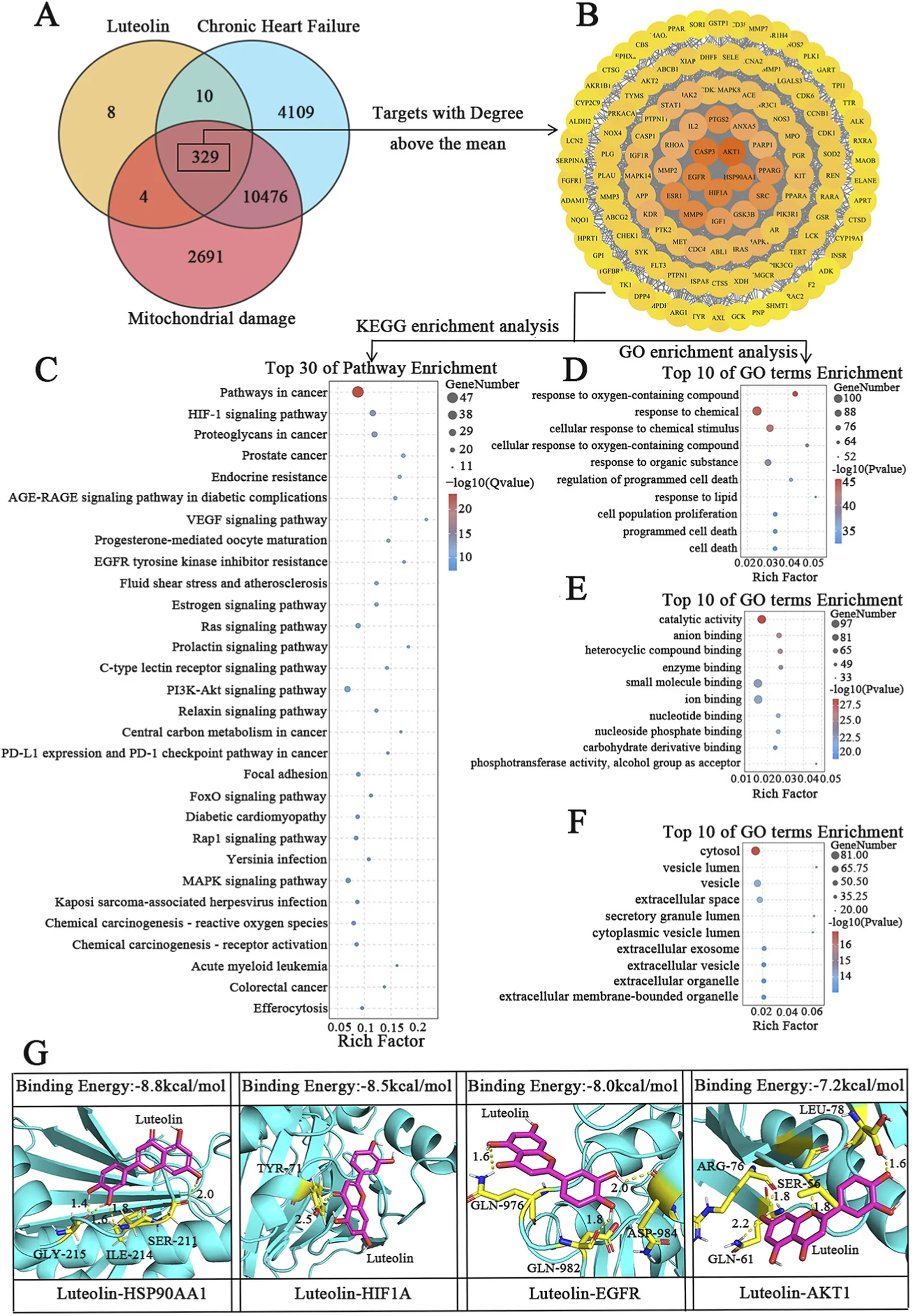
Network pharmacology analysis and molecular docking. **(A)** Venn diagram showing the overlapping targets among luteolin, mitochondrial damage, and CHF (329 common targets). **(B)** PPI network of core targets (degree above the mean). **(C)** Top 30 significantly enriched pathways from KEGG enrichment analysis of core targets. **(D)** Top 10 enriched biological processes from GO enrichment analysis. **(E)** Top 10 enriched molecular functions from GO enrichment analysis. **(F)** Top 10 enriched cellular components from GO enrichment analysis. **(G)** Molecular docking showing the binding modes of luteolin with HSP90AA1 (−8.8 kcal/mol), HIF1A (−8.5 kcal/mol), EGFR (−8.0 kcal/mol), and AKT1 (−7.2 kcal/mol).

### 3.2 Screening of the L-M-C gene set and validation of the clinical prediction model

The Lasso + RF combination was identified as the optimal predictive model, with the highest average AUC of 0.749 (Figure 3A). This model included six significant genes: HIF1A, NOS2, BNIP3, BNIP3L, PDK1, and BBC3, collectively termed the L-M-C gene set. A PPI network generated for the proteins encoded by these six genes showed strong interactions among five genes, while BBC3 was not connected in the network (Figure 3D). Correlation analysis of the expression profiles within the L-M-C gene set showed that HIF1A was strongly positively correlated with BNIP3, BNIP3L, and PDK1 (Figure 3C).

**FIGURE 3:**
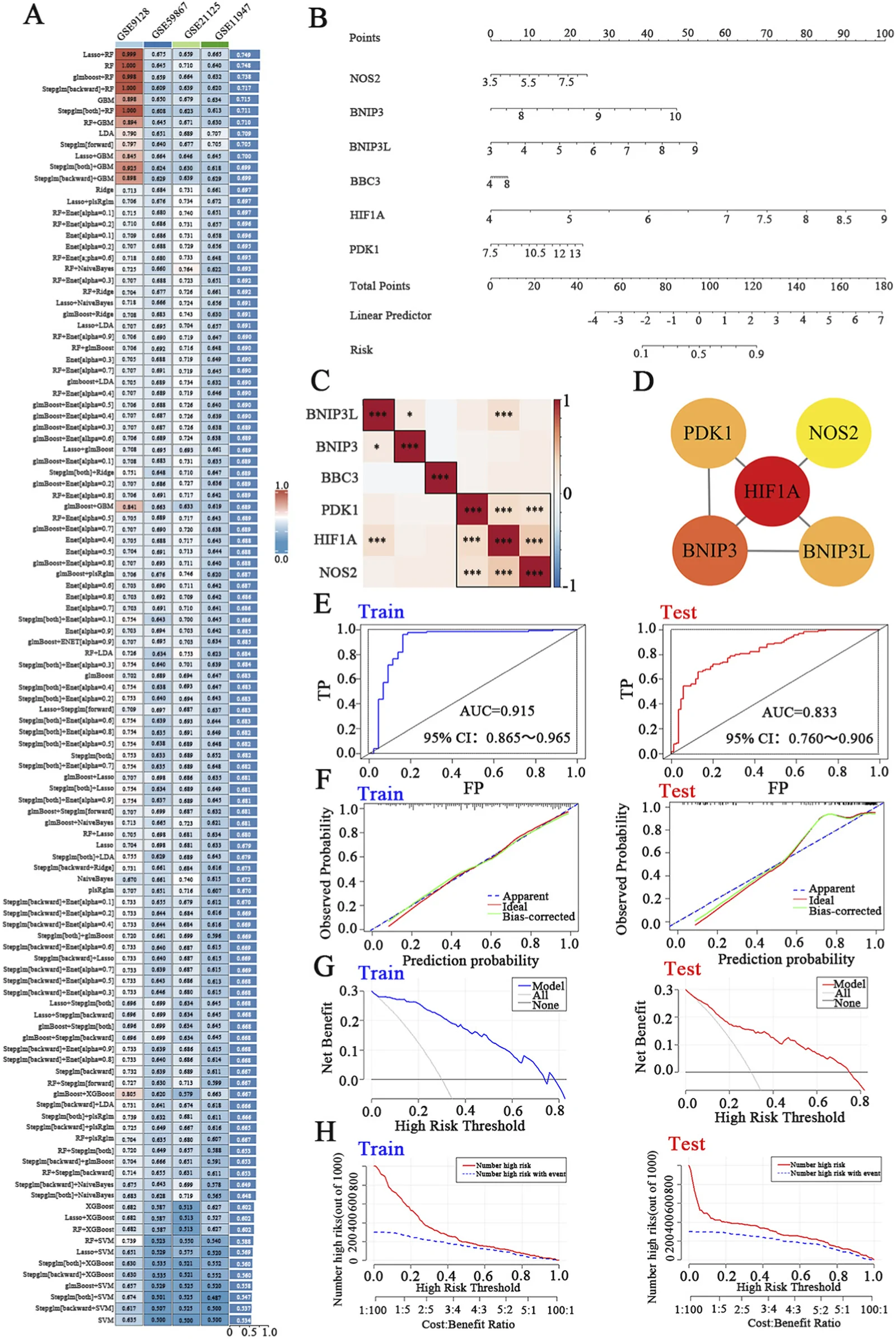
Screening of the L-M-C gene set and validation of the clinical prediction model. **(A)** AUC values of 113 algorithm combinations across four datasets (GSE9128, GSE59867, GSE21125, GSE11947), with Lasso + RF ranked first. **(B)** Nomogram constructed using the L-M-C gene set from the training cohort. **(C)** Correlation heatmap of the six L-M-C genes. *: *P* < 0.05; ***: *P* < 0.001. **(D)** PPI network of the L-M-C gene set (BBC3 was not connected in the network). **(E)** ROC curves of the L-M-C gene set in the training (AUC = 0.915) and validation (AUC = 0.833) cohorts. **(F)** Calibration curves of the L-M-C gene set in the training and validation cohorts. **(G)** DCA curves of the L-M-C gene set in the training and validation cohorts. **(H)** CIC curves of the L-M-C gene set in the training and validation cohorts.

To further evaluate the predictive performance of the Lasso + RF model, a nomogram was constructed using R to visualize the relationship between the L-M-C gene set and CHF progression. Within the nomogram, each feature variable was assigned a specific point value, and the total score corresponded to the probability of CHF (Figure 3B). ROC curve analysis revealed an AUC of 0.915 for the training cohort and 0.833 for the independent validation cohort (Figure 3E). Additionally, the calibration curve confirmed good calibration of this nomogram model for CHF (Figure 3F). Both decision curve analysis (DCA) and clinical impact curves (CIC) further confirmed the good predictive performance of the nomogram (Figures 3G,H).

### 3.3 Construction of the single-cell transcriptomic atlas for CHF and dynamic expression analysis of the L-M-C gene set

In the scRNA-seq dataset, the samples exhibited successful integration with no discernible batch effects, validating the dataset for subsequent analyses. Based on the transcriptomic signatures of canonical marker genes, all cells were clustered into 21 distinct clusters and annotated into 8 major cell types: Fibroblasts, Endothelial cells, Smooth muscle cells, Cardiomyocytes, Macrophages, Neutrophils, NK cells, and Neuronal cells. Compared with the control group, the proportions of NK cells and macrophages were significantly increased in the heart failure group, whereas the proportions of smooth muscle cells and neuronal cells were significantly decreased (Figures 4A–D). Additionally, the proportional distribution of each cell lineage across the two samples was evaluated (Figure 4F). Using the AddModuleScore function to evaluate the L-M-C gene set across these distinct lineages, the L-M-C score was found to be significantly higher in Cardiomyocytes compared to all other cell types (Figure 4E).

**FIGURE 4:**
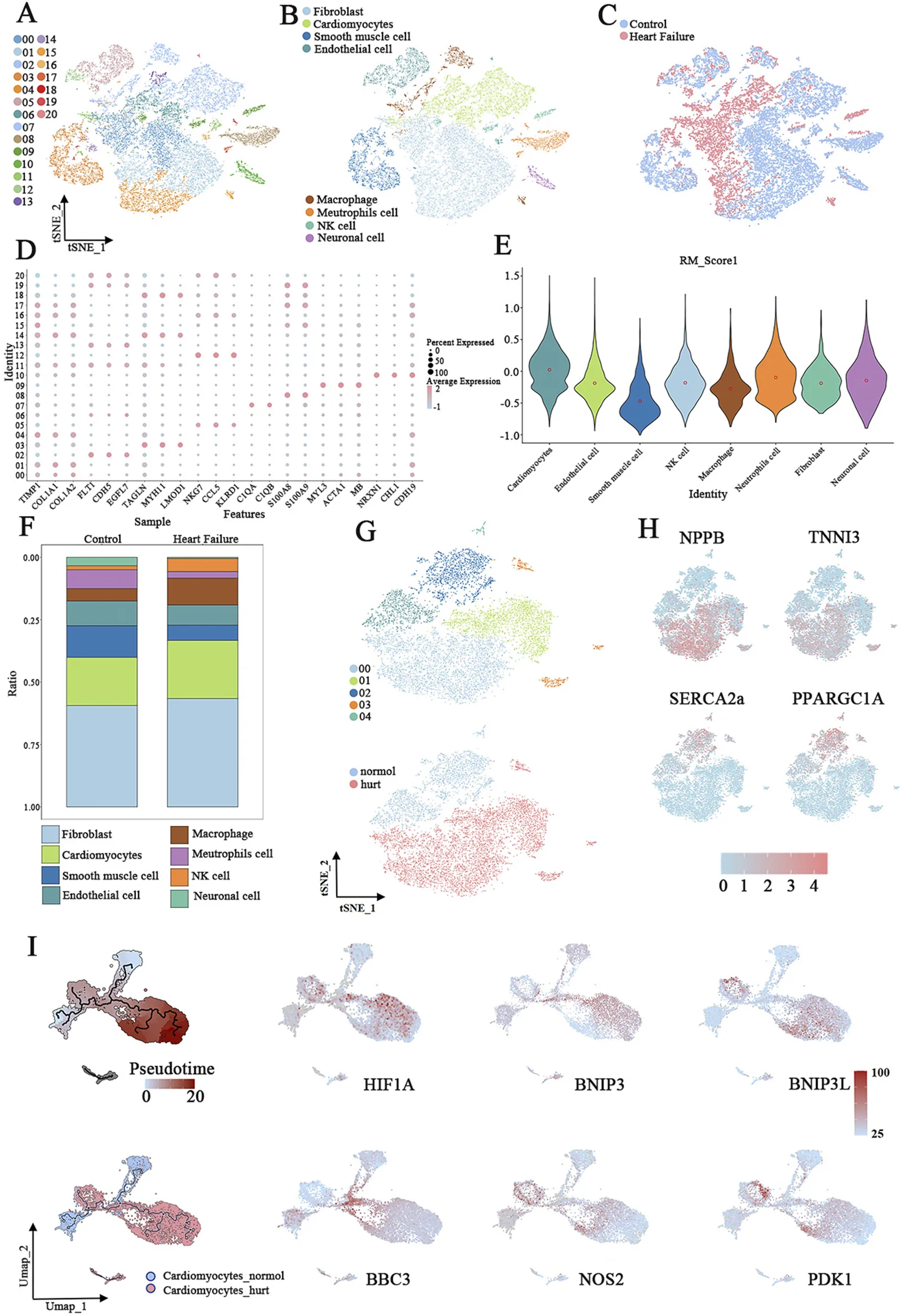
Single-cell transcriptomic analysis of the CHF myocardium. **(A)** t-SNE plot showing 21 cell clusters. **(B)** t-SNE plot annotated with eight cell types (fibroblasts, cardiomyocytes, smooth muscle cells, endothelial cells, macrophages, neutrophils, NK cells, and neuronal cells). **(C)** t-SNE plot colored by group (Control vs. Heart Failure). **(D)** Dot plot showing the expression of canonical marker genes across cell clusters. **(E)** Violin plot showing L-M-C module scores across cell types, with the highest score in cardiomyocytes. **(F)** Stacked bar chart showing the proportional distribution of each cell type in the Control and Heart Failure groups. **(G)** t-SNE plot of cardiomyocyte subpopulations, divided into normal and injured (hurt) states. **(H)** Expression of cardiomyocyte subtype markers (NPPB, TNNI3, SERCA2a, and PPARGC1A) in cardiomyocyte subpopulations. **(I)** Pseudotime trajectory analysis showing the expression dynamics of the six L-M-C genes during the transition from normal to injured cardiomyocytes.

Given this pronounced L-M-C score elevation, the annotated Cardiomyocyte subpopulation was isolated for further analysis. Based on marker gene expression, the Cardiomyocytes were further divided into normal Cardiomyocytes (normal), characterized by high expression of SERCA2a and PPARGC1A, and injured Cardiomyocytes (hurt), characterized by elevated expression of NPPB and TNNI3 (Figures 4G,H). Analysis of L-M-C gene expression along the pseudotime trajectory from the “normal” to “hurt” cluster was performed. The analysis revealed that the expression levels of the genes in the L-M-C gene set (HIF1A, NOS2, BNIP3, BNIP3L, PDK1, and BBC3) progressively increased with the transition in cellular state (Figure 4I).

### 3.4 CCK-8 screening for optimal luteolin concentration

The results indicated that after 24 h of treatment, luteolin at 5, 10, 15, and 20 μmol/L showed no significant effect on the inhibition rate of H9C2 cells compared to the Control group. At 48 h, the 5 μmol/L, 10 μmol/L, and 15 μmol/L concentrations showed no significant effect, whereas 20 μmol/L significantly increased the inhibition rate (*P* < 0.05). At 72 h, 5 μmol/L and 10 μmol/L concentrations showed no significant effect on the inhibition rate, but both 15 μmol/L and 20 μmol/L significantly increased the inhibition rate (*P* < 0.05) (Figure 5A). Consequently, 10 μmol/L—the highest concentration showing no significant cytotoxicity in H9C2 cells at 72 h—was selected as the optimal concentration for subsequent experiments.

**FIGURE 5:**
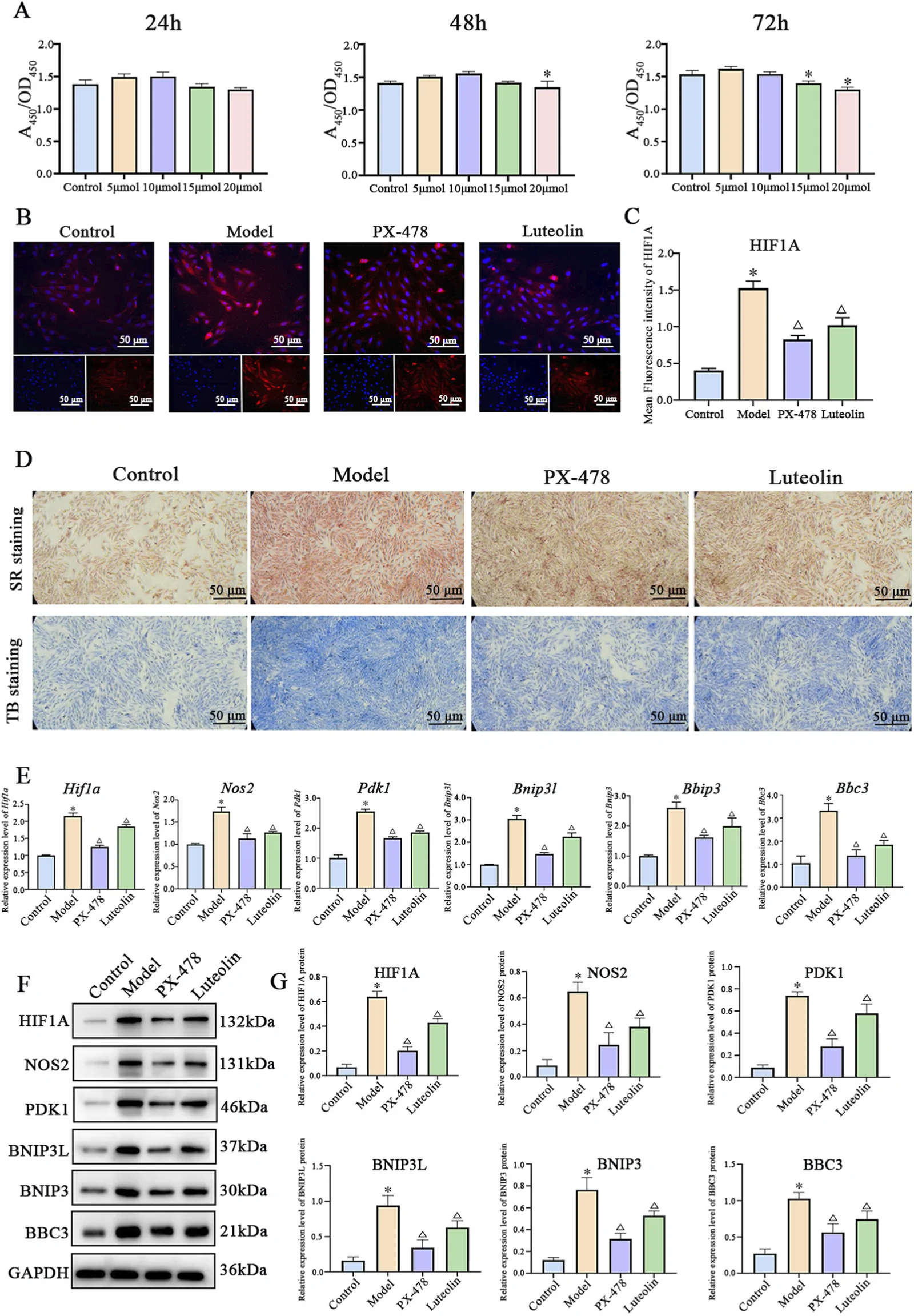
*In vitro* experimental results. **(A)** Effect of different luteolin concentrations on H9C2 cell viability at 24, 48, and 72 h (CCK-8 assay). **(B)** Immunofluorescence images of HIF1A in H9C2 cells (200×). **(C)** Quantitative analysis of mean HIF1A fluorescence intensity across groups. **(D)** Toluidine Blue (TB) and Sirius Red (SR) staining of H9C2 cells across all experimental groups (200×). **(E)** Relative mRNA expression of the six L-M-C genes. **(F)** Western blot bands of the six L-M-C proteins. **(G)** Relative protein expression of the six L-M-C genes. n = 3; *: *P* < 0.05 vs. Control group; Δ: *P* < 0.05 vs. Model group.

### 3.5 Luteolin downregulates HIF1A protein fluorescence expression in heart failure cardiomyocytes

In the Control group, HIF1A immunofluorescence in cardiomyocytes showed weak baseline fluorescence with a low proportion of positive cells, indicating minimal constitutive expression of HIF1A. In contrast, the Model group exhibited a markedly increased HIF1A signal, with strong red fluorescence in most cells, indicative of high HIF1A expression. Following intervention with PX-478 or Luteolin, the HIF1A fluorescence signal was substantially attenuated relative to the Model group, reaching an intermediate level. These findings visually confirm that both PX-478 and Luteolin effectively suppressed HIF1A overexpression (Figure 5B). Quantitative analysis of the mean fluorescence intensity confirmed that the Model group had significantly higher HIF1A expression than the Control group (*P* < 0.05), whereas the PX-478 and Luteolin groups showed significantly lower mean fluorescence intensity than the Model group (*P* < 0.05) (Figure 5C).

### 3.6 Luteolin alleviates the severity of fibrosis in model cardiomyocytes

Toluidine Blue Staining: H9C2 cells in the Control group showed uniform pale staining without localized dark-stained areas, indicating a low fibrotic tendency. Cells in the Model group showed markedly intensified staining with prominent dark-stained regions surrounding the cytoplasm and nuclei, indicating a strong fibrotic tendency. Compared with the Model group, both the PX-478 and Luteolin groups showed lighter staining with fewer dark-stained areas, indicating decreased production of glycosaminoglycans (GAGs) and significant attenuation of the fibrotic tendency.

Sirius Red Staining: Cells in the Control group showed pale staining with virtually no dark staining, reflecting minimal fibrosis. The Model group showed intense staining with dense red coloration across extensive fields of view, indicating severe fibrosis. The PX-478 and Luteolin groups showed notably paler staining with smaller areas of intense staining compared with the Model group, indicating significant attenuation of fibrotic severity (Figure 5D).

### 3.7 Luteolin reduces the mRNA expression levels of the L-M-C gene set

Compared with the Control group, the mRNA expression levels of *Hif1a*, *Nos2*, *Bnip3*, *Bnip3l*, *Pdk1* and *Bbc3* in H9C2 cells of the Model group were significantly upregulated (*P* < 0.05). Compared with the Model group, treatment with either the HIF-1 inhibitor or luteolin significantly downregulated the mRNA expression of all six L-M-C genes (*P* < 0.05) (Figure 5E).

### 3.8 Luteolin decreases the protein expression levels of the L-M-C gene set

Consistent with the mRNA expression trends, the protein expression levels of HIF1A, NOS2, BNIP3, BNIP3L, PDK1, and BBC3 were significantly elevated in the Model group compared with the Control group (*P* < 0.05). Compared with the Model group, treatment with either the inhibitor or luteolin significantly reduced the protein expression of all six genes in H9C2 cells (*P* < 0.05) (Figures 5F,G).

### 3.9 Luteolin ameliorates myocardial histopathological damage and mitigates myocardial fibrosis

H&E Staining: The Control group showed highly organized cardiomyocyte alignment with intact structural integrity. The Model group, however, exhibited disorganized cellular arrangements, hyperchromatic cytoplasm, nuclear pyknosis, karyolysis, and focal necrotic lesions. The Luteolin and Sacubitril/Valsartan groups showed improved structural organization with significantly reduced cellular injury and necrosis.

Masson’s Trichrome Staining: The myocardial interstitium of the Control group contained only sparse collagen fibers. The Model group showed extensive proliferation and deposition of collagen fibers. Interventions with Luteolin and Sacubitril/Valsartan significantly reduced collagen proliferation compared with the Model group.

Sirius Red Staining: Under bright-field microscopy, the Control group myocardium was faintly stained with negligible fibrosis, while the Model group displayed extensive deep-red staining, indicating severe fibrosis. The drug-intervention groups showed a markedly reduced area of red staining. Under polarized light, the Control group contained only trace amounts of type III collagen. The Model group showed abundant dense, interwoven type I and type III collagen. Treatment with Luteolin and Sacubitril/Valsartan effectively reduced the abnormal deposition of both collagen types, thereby ameliorating myocardial fibrosis (Figure 6A).

**FIGURE 6:**
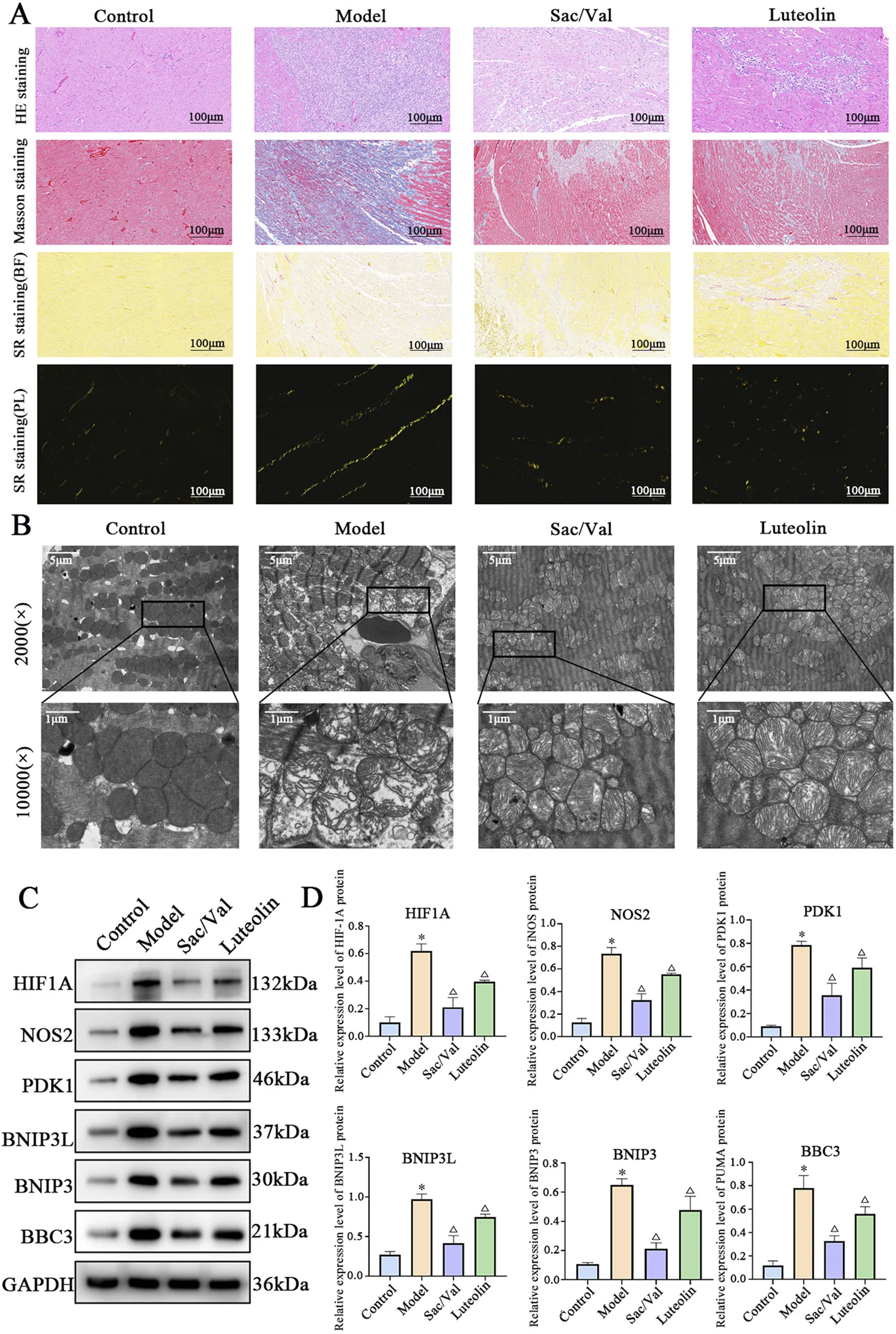
Histological staining, transmission electron microscopy, and Western blot results. **(A)** Histological staining of myocardial tissues across all rat groups, including H&E staining (row 1), Masson’s trichrome staining (row 2), Sirius Red staining under bright field (row 3), and Sirius Red staining under polarized light (row 4). Scale bar = 100 μm. **(B)** Transmission electron microscopy of myocardial mitochondrial ultrastructure across all rat groups. Upper row: ×2000; lower row: magnified view at ×10000. **(C)** Western blot bands of the six L-M-C proteins in myocardial tissues. **(D)** Relative protein expression of the six L-M-C genes across all rat groups. n = 3; *: *P* < 0.05 vs. Control group; Δ: *P* < 0.05 vs. Model group.

### 3.10 Luteolin attenuates myocardial mitochondrial ultrastructural damage in CHF rats

Transmission electron microscopy revealed that the myocardial mitochondria in the Control group showed a regular oval morphology, intact outer membranes, and densely packed, highly ordered cristae. In contrast, mitochondria in the Model group were extensively swollen and deformed, displaying ruptured membranes, extensive dissolution and disruption of cristae, and severe vacuolization. In the Sacubitril/Valsartan and Luteolin groups, mitochondrial swelling was significantly attenuated, membrane integrity was largely preserved, and cristae depletion and vacuolization were significantly reduced, indicating effective rescue of mitochondrial ultrastructural damage (Figure 6B).

### 3.11 Luteolin reduces the protein expression levels of the L-M-C gene set in the myocardium of CHF rats

Compared with the Control group, myocardial tissues of the Model group showed significantly increased protein expression of HIF1A, NOS2, BNIP3, BNIP3L, PDK1, and BBC3 (*P* < 0.05). Compared with the Model group, the Sacubitril/Valsartan and Luteolin groups showed significantly reduced expression of these L-M-C proteins in cardiac tissues (*P* < 0.05) (Figures 6C,D).

### 3.12 Luteolin decreases the cardiomyocyte apoptosis rate in CHF rats

Compared with the control group, myocardial tissues of the model group showed a significantly higher proportion of TUNEL-positive cells. Furthermore, the mean fluorescence intensity of HIF1A and Caspase-3 was significantly increased, along with a significantly elevated pro-apoptotic Bax/anti-apoptotic Bcl-2 ratio (*P* < 0.05). Compared with the model group, the sacubitril/valsartan and luteolin groups showed a significantly lower proportion of TUNEL-positive cells, reduced mean fluorescence intensity of HIF1A and Caspase-3, and a significantly decreased Bax/Bcl-2 ratio (P < 0.05) (Figures 7A–F).

**FIGURE 7:**
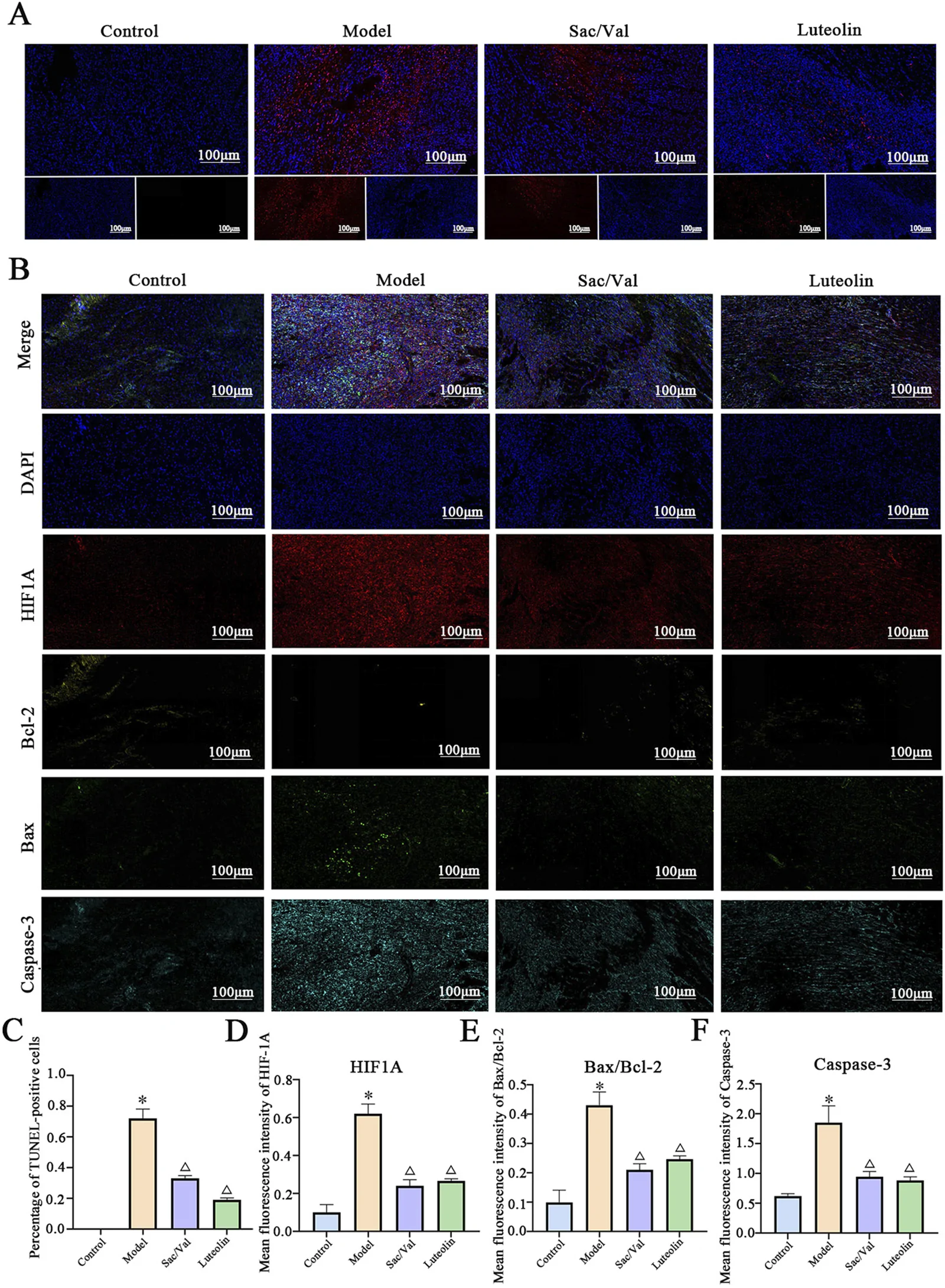
TUNEL staining and immunofluorescence results. **(A)** TUNEL staining of myocardial tissue sections across all rat groups. Scale bar = 100 μm. **(B)** Immunofluorescence staining of HIF-1A, Bcl-2, Bax, and Caspase-3 in myocardial tissue sections across all rat groups. Scale bar = 100 μm. **(C)** Percentage of TUNEL-positive cells across groups. **(D)** Mean fluorescence intensity of HIF-1A across groups. **(E)** Mean fluorescence intensity ratio of Bax/Bcl-2 across groups. **(F)** Mean fluorescence intensity of Caspase-3 across groups. n = 3; *: *P* < 0.05 vs. Control group; Δ: *P* < 0.05 vs. Model group.

## 4 Discussion

Cardiomyocyte apoptosis induced by the structural and functional impairment of mitochondria constitutes a central pathological mechanism driving the onset and progression of chronic heart failure (CHF) (Pavlović et al., 2025; Hinton et al., 2024; Chen et al., 2026). As an organ with high energy demands, the myocardium relies heavily on mitochondrial aerobic metabolism. Consequently, compromised mitochondrial membranes, fragmented cristae, and dysregulated energy metabolism lead to cardiomyocyte apoptosis, abnormal myocardial collagen deposition, and ventricular remodeling, which collectively drive the progressive deterioration of cardiac function (Fang and Gustafsson, 2026; Brunel-Guitton et al., 2015). HIF1A, serving as the core oxygen-responsive subunit of the HIF-1 signaling pathway, aberrantly accumulates under chronic myocardial hypoxia. By transcriptionally modulating downstream target genes, it disrupts mitophagy, glucolipid metabolism, and oxidative stress homeostasis, thereby acting as a pivotal molecular hub that mediates hypoxia-induced myocardial mitochondrial injury (Sato et al., 2023; Qiliqiangxin, 2023). Luteolin is a naturally occurring flavonoid compound ubiquitously found in edible and medicinal plants. Previous investigations have firmly established its remarkable pharmacological activities—including potent antioxidant effects, the attenuation of myocardial fibrosis, and the restoration of myocardial energy metabolism—highlighting its distinct therapeutic potential in the realm of cardiovascular diseases associated with mitochondrial damage (Pan et al., 2025; Luo et al., 2017). Capitalizing on network pharmacology screening, the present study identified 329 overlapping target genes shared among luteolin, mitochondrial damage, and CHF. GO enrichment analysis revealed that these overlapping targets were mainly enriched in processes such as cell death and programmed cell death. Concurrently, KEGG enrichment analysis revealed that the overlapping target genes were significantly enriched in multiple signaling pathways, including the HIF-1 signaling pathway, VEGF signaling pathway, and Ras signaling pathway, suggesting that luteolin may exert its anti-chronic heart failure effect through the synergistic regulation of multiple pathways. Among them, the HIF-1 signaling pathway ranked second in enrichment significance among all pathways. These bioinformatics insights provide preliminary evidence that luteolin may exert its anti-CHF efficacy by selectively modulating the HIF-1 signaling pathway to alleviate mitochondrial damage and subsequently inhibit cardiomyocyte apoptosis. Using 113 machine learning algorithm combinations, we screened these core targets to identify an L-M-C gene signature consisting of HIF1A, NOS2, BNIP3, BNIP3L, PDK1, and BBC3. A clinical nomogram prediction model for CHF based on these six genes demonstrated favorable predictive performance, underscoring the strong potential of this gene combination in predicting early CHF risk. Single-cell transcriptomic analyses further confirmed these findings: within the single-cell atlas of the CHF myocardium, the module score of the L-M-C gene set was significantly higher in the cardiomyocyte subpopulation compared with other cell lineages. Pseudotime trajectory analysis showed that the pathological transition from normal to damaged cardiomyocytes was accompanied by a progressive increase in the expression of the six L-M-C genes. Subsequent *in vitro* and *in vivo* experimental validations confirmed the critical involvement of the L-M-C gene set in the pathogenesis of mitochondrial damage and CHF, thereby confirming the reliability of our bioinformatics predictions.

Among the six target genes encompassing the L-M-C signature, HIF1A functions as the master regulator of the HIF-1 signaling pathway. With the exception of BBC3, the remaining genes—NOS2, BNIP3, BNIP3L, and PDK1—are direct downstream targets of the HIF-1 signaling pathway. While previous studies have independently verified the involvement of these individual genes in myocardial mitochondrial injury and the pathology of heart failure, no previous study has integrated them into a composite biomarker panel to decipher CHF mechanisms or predict clinical risk. Previous studies have shown that HIF1A, hyperactivated by hypoxia, transcriptionally upregulates BNIP3 and BNIP3L. As key receptors for mitophagy, their anomalous overexpression triggers excessive mitochondrial autophagy, leading to the massive intracellular accumulation of defective mitochondria, which significantly exacerbates necrotic and apoptotic cardiomyocyte death (Diwan, 2024). Upon regulation by HIF1A, PDK1 inhibits pyruvate dehydrogenase activity, driving the failing myocardium to shift from fatty acid oxidation to inefficient glycolysis. This disruption of myocardial energetic homeostasis is a hallmark molecular event of metabolic remodeling in heart failure (Kim et al., 2006). The anomalous overexpression of inducible nitric oxide synthase (NOS2) catalyzes the excessive production of nitric oxide, culminating in severe myocardial oxidative stress and lipid peroxidation damage to mitochondrial membranes (Pall, 2013). BBC3 (also known as PUMA), a classical pro-apoptotic gene, plays a critical role in the pathological cascade of mitochondrial impairment. As a key pro-apoptotic member of the BH3-only protein family, upregulated BBC3 translocates to the mitochondria, instigating the collapse of the mitochondrial membrane potential, cristae fragmentation, extensive reactive oxygen species (ROS) accumulation, and outer membrane rupture. This mediates mitochondria-dependent apoptosis, positioning BBC3 as a key mediator of irreversible myocardial mitochondrial damage under stress conditions (Yu and Zhang, 2008; Chipuk and Green, 2009). Furthermore, different regulatory mechanisms exist among the six molecules comprising the L-M-C gene set. HIF1A acts as the core transcription factor responsive to hypoxia (Sato et al., 2023; Qiliqiangxin, 2023). The promoters of the NOS2, BNIP3, BNIP3L, and PDK1 genes harbor Hypoxia Response Elements (HREs) and are therefore directly transcriptionally activated by stabilized HIF-1α (Da Dalt et al., 2023; Muruganathan et al., 2022). Specifically, BNIP3 and BNIP3L—bifunctional proteins governing both mitophagy and apoptosis—can directly increase mitochondrial membrane permeability, triggering intrinsic myocardial apoptosis (Lopaschuk et al., 2021; Nordén et al., 2025). Concurrently, NOS2 indirectly exacerbates mitochondrial structural damage by inducing oxidative stress through excessive NO production (Pall, 2013). Conversely, the pro-apoptotic gene BBC3 lacks an HRE binding site and is not directly regulated by HIF-1α; its expression is upregulated only after extensive mitochondrial damage and ROS accumulation, serving to synergistically amplify apoptotic myocardial injury (Yu and Zhang, 2008; Chipuk and Green, 2009). Notably, although BBC3 is not directly transcriptionally regulated by HIF-1α, our experimental data demonstrated that inhibition of the HIF-1 pathway by PX-478 or luteolin consistently downregulated BBC3 at both the mRNA and protein levels. This apparent discrepancy can be explained by an indirect regulatory axis: HIF-1α activation promotes mitochondrial damage and ROS accumulation, which in turn stabilizes and activates p53; BBC3, as a well-established p53 transcriptional target, is consequently upregulated. In this model, HIF-1α inhibition reduces ROS burden, attenuates p53 activation, and thereby secondarily suppresses BBC3 expression. However, this hypothesized ROS/p53/BBC3 axis has not been experimentally validated in the present study, and future studies using p53 inhibitors or ROS scavengers in rescue experiments are warranted to delineate this indirect regulatory pathway. Existing literature has predominantly investigated the isolated roles of individual genes in myocardial injury, leaving a notable void in exploring the correlation between coordinated multigenic expression patterns and CHF pathogenesis. Using multi-algorithm machine learning, this study integrated six functionally related genes crucial to mitochondrial damage into the L-M-C gene set. By establishing a clinical prediction model, we provide a tool for predicting CHF risk based on this signature, elucidating the molecular mechanisms underpinning luteolin’s therapeutic intervention in CHF and thereby providing new therapeutic targets for natural flavonoids in heart failure management.

The present *in vitro* and *in vivo* investigations confirm the pharmacological mechanism by which luteolin treats CHF: by regulating mitochondrial damage to suppress cardiomyocyte apoptosis. In both the doxorubicin-induced H9C2 cardiomyocyte injury model and the coronary artery ligation-induced rat CHF model, pathological indices associated with myocardial damage were significantly altered in the Model groups. *In vitro*, the mRNA and protein expression of the L-M-C genes increased significantly, while Toluidine blue and Sirius red staining revealed aggravated cellular collagen deposition and fibrotic progression. *In vivo*, the CHF rats exhibited marked ventricular dilation (significantly elevated LVEDd and LVESd) alongside a significant reduction in cardiac function (significantly reduced LVEF and LVFS). H&E, Masson’s trichrome, and Sirius red staining collectively confirmed disorganized cardiomyocyte architectures and the pervasive, interwoven deposition of type I and type III collagen. Transmission electron microscopy revealed severe mitochondrial swelling, rupture, cristae dissolution, and vacuolization. Furthermore, HIF1A and the apoptotic marker Caspase-3 were both markedly upregulated in the myocardial tissues of CHF rats, alongside a significant elevation in the ratio of pro-apoptotic Bax to anti-apoptotic Bcl-2. Treatment with the HIF-1 signaling pathway inhibitor PX-478 remarkably reversed the heightened expression of the L-M-C genes in model cells and mitigated fibrotic cellular injury, confirming that inhibiting the HIF-1 pathway can effectively alleviate myocardial mitochondrial damage. Concurrently, luteolin treatment at 10 μmol/L showed therapeutic effects comparable to those of PX-478, significantly downregulating the expression of the six target genes within the L-M-C set and restoring normal cellular morphology. *In vivo*, Sacubitril/Valsartan—a first-line clinical medication for heart failure—was employed as a positive control. Following 28 days of luteolin administration via gastric gavage, the cardiac functional parameters of the rats rebounded notably. Myocardial histopathological lesions, collagen deposition, and mitochondrial ultrastructural damage were concurrently ameliorated. Correspondingly, the expression levels of the L-M-C genes and apoptosis-related proteins decreased significantly relative to the Model group, demonstrating therapeutic efficacy comparable to that of the positive control drug sacubitril/valsartan. Collectively, these results confirm that luteolin exerts therapeutic effects against CHF by suppressing the HIF-1 signaling pathway and the subsequent expression of the L-M-C gene set, thereby mitigating mitochondrial damage and inhibiting cardiomyocyte apoptosis.

Although this study has demonstrated the role of luteolin in regulating the HIF1A/L-M-C axis to ameliorate mitochondrial damage and treat CHF, several limitations remain. First, the study used only pharmacological intervention to verify luteolin’s regulation of the L-M-C gene set, and lacked gene-editing validation, including lentivirus-mediated overexpression or AAV-mediated myocardium-specific HIF1A knockout *in vitro* and *in vivo*. Second, all bioinformatics data were derived from public GEO microarray datasets. The nomogram was trained on GSE9128 and validated on the integrated GSE59867/GSE21125/GSE11947 cohorts. Although all datasets enrolled heart failure patients, heterogeneity existed across datasets in terms of etiology, cardiac functional classification, and control definitions—some controls may include myocardial infarction or left ventricular dysfunction—which may affect L-M-C gene screening and model stability. Furthermore, no independent clinical cohort or CHF patient samples were used for external validation, limiting the generalizability of the prediction model. Future multicenter prospective validation is warranted. Third, critical mediators of the mitochondrial apoptotic cascade were not assessed; the molecular regulation linking mitochondrial damage to apoptosis awaits further elucidation. Lastly, network pharmacology identified AKT1 and EGFR as additional core PPI network nodes in CHF, but these targets were not selected for in-depth validation. Future functional validation of other core PPI nodes is needed to address this gap.

## 5 Conclusion

In summary, using integrated methodologies including network pharmacology, machine learning, single-cell transcriptomics, and *in vitro* and *in vivo* experiments, this study systematically validates the mechanism by which luteolin treats CHF. By inhibiting the HIF-1 signaling pathway and downregulating the expression of the L-M-C gene set, luteolin effectively alleviates structural and functional damage of myocardial mitochondria and suppresses cardiomyocyte apoptosis. These findings enrich the molecular mechanistic understanding of natural flavonoids in the treatment of CHF, providing a scientific basis for the clinical application and drug development of luteolin.

## Data Availability

The datasets presented in this study can be found in online repositories. The names of the repository/repositories and accession number(s) can be found in the article/Supplementary Material.
