# Supplementary material for: Mechanism of luteolin in treating chronic heart failure by regulating HIF-1 signaling pathway-mediated mitochondrial damage to inhibit cardiomyocyte apoptosis
Source: Front Cell Dev Biol. 2026 Oct 2;14:1977166. doi: 10.3389/fcell.2026.1977166 (PMC13672322; doi:10.3389/fcell.2026.1977166)
Supplement: Supplementary file 1 [file Table1.docx]

**Table S1 Primer sequences for RT-qPCR**

| **Primer name** | **Primer sequence (5′‑3′)** | **Length** |
| --- | --- | --- |
| Rat-*Hif1a*-F | CCTCTGGACTTGCCCCTTTC | 162bp |
| Rat-*Hif1a*-R | CGACGTTCGGAACTCATCCT |  |
| Rat-*Bnip3*-F | TCGCTCCCAGACACCACA | 164bp |
| Rat-*Bnip3*-R | GCCGACTTGACCAATCCC |  |
| Rat-*Bbc3*-F | ACCAGCCCAGCAGCACCTAG | 178bp |
| Rat-*Bbc3*-R | TTGTCTCCGCCGCTCGTAC |  |
| Rat-*Nos2*-F | CACAGTGTCGCTGGTTTGAA | 148bp |
| Rat-*Nos2*-R | TGTGCTTCTGCACCAACTCT |  |
| Rat-*Pdk1*-F | TCGGTATGAGGCTGGCAAGG | 185bp |
| Rat-*Pdk1*-R | CTTCATGGAGAGTGGCGACG |  |
| Rat-*Bnip3l*-F | GGCAACGGTAATGGAAAT | 163bp |
| Rat-*Bnip3l*-R | CTTGTGGTGAAGGGCTGT |  |
| Rat-*Gapdh*-F | TGTCTCCTGCGACTTCAACA | 182bp |
| Rat-*Gapdh*-R | GGTGGTCCAGGGTTTCTTACT |  |

**Table S2 Comparison of cardiac function in rats between control and model groups(‾x±s ,n=5)**

| **Group** | LVEDd(mm) | LVESd(mm) | LVEF(%) |
| --- | --- | --- | --- |
| Control group | 4.14±0.28 | 1.82±0.15 | 85.55±8.90 |
| Model group | 8.33±1.29* | 7.06±0.95* | 39.05±3.96* |

Note: Compared with the control group, ^*^*P*＜0.05.
